# Caregiver and youth self-reported emotional and behavioural problems in Ugandan HIV-infected children and adolescents

**DOI:** 10.4102/sajpsychiatry.v24i0.1265

**Published:** 2018-09-20

**Authors:** Leigh L. van den Heuvel, Jonathan Levin, Richard S. Mpango, Kenneth D. Gadow, Vikram Patel, Jean B. Nachega, Soraya Seedat, Eugene Kinyanda

**Affiliations:** 1 Department of Psychiatry, Faculty of Medicine and Health Sciences, Stellenbosch University, South Africa; 2 School of Public Health, Faculty of Health Sciences, University of the Witwatersrand, South Africa; 3 Mental Health Project, MRC/UVRI Uganda Research Unit on AIDS/Senior Wellcome Trust Fellowship, Entebbe, Uganda; 4 Department of Psychiatry, Stony Brook University, United States; 5 Department of Global Health and Social Medicine, Harvard Medical School, Massachusetts, United States; 6 Departments of Epidemiology, Infectious Diseases and Microbiology, University of Pittsburgh Graduate School of Public Health, United States

## Abstract

**Introduction:**

We determined the prevalence of, and factors associated with, self-rated emotional and behavioural problems (EBPs) and assessed the agreement between self-rated and caregiver-rated EBPs in the ‘Mental health among HIV-infected Children and Adolescents (CA-HIV) in Kampala and Masaka, Uganda’ (CHAKA) study. Existing literature demonstrates that CA-HIV face increased mental health challenges related to a broad range of biological and psychosocial factors. There is scarce data on self-reported EBPs in CA-HIV.

**Methods:**

In a cross-sectional sample, caregiver-reported EBPs were assessed with the Child and Adolescent Symptom Inventory-5 (CASI-5), and self-reported problems were evaluated with the Youth Inventory-4 (YI-4) in 469 adolescents aged 12–17 years and the Child Inventory-4 (CI-4) in 493 children aged 8–11 years. Logistic regression models were utilised to determine factors related to self-reported EBPs.

**Results:**

Self-reported emotional problems (EPs) were present in 28.8% of the adolescents and were associated with caregivers being separated and having a lower level of education. Among adolescents, 14.5% had self-reported behavioural problems (BPs), and these were associated with caregiver unemployment and food insecurity. Self-reported EPs were reported by 36.9% of children and were associated with rural study sites, having missed school and caregivers having a lower level of education. There was only modest agreement (maximum *r* = 0.29) between caregiver- and CA-HIV-reported EBPS, with caregivers reporting more EPs and adolescents reporting more BPs.

**Conclusion:**

Self-reported EBPs are frequently endorsed by CA-HIV, and these problems are related to unique psychosocial factors. Including CA-HIV, self-report measures can assist in identifying problems that caregivers may not be aware of, particularly BPs.

